# Composition and structure of the parasite faunas of cod, *Gadus morhua *L. (Teleostei: Gadidae), in the North East Atlantic

**DOI:** 10.1186/1756-3305-1-23

**Published:** 2008-07-18

**Authors:** Diana Perdiguero-Alonso, Francisco E Montero, Juan Antonio Raga, Aneta Kostadinova

**Affiliations:** 1 Marine Zoology Unit, Cavanilles Institute of Biodiversity and Evolutionary Biology, University of Valencia, PO Box 22085, 46071, Valencia, Spain; 2 Department of Animal Biology, Plant Biology and Ecology, Autonomous University of Barcelona, Campus Universitari, 08193, Bellaterra, Barcelona, Spain; 3 Central Laboratory of General Ecology, Bulgarian Academy of Sciences, 2 Gagarin Street, 1113, Sofia, Bulgaria

## Abstract

**Background:**

Although numerous studies on parasites of the Atlantic cod, *Gadus morhua *L. have been conducted in the North Atlantic, comparative analyses on local cod parasite faunas are virtually lacking. The present study is based on examination of large samples of cod from six geographical areas of the North East Atlantic which yielded abundant baseline data on parasite distribution and abundance.

**Materials and Methods:**

A total of 826 fish was sampled in the Baltic, Celtic, Irish and North seas, Icelandic waters and Trondheimsfjord (Norway) in 2002 (spring and autumn) and 2003 (spring). The gills and internal organs (oesophagus, stomach, intestine, pyloric caeca, liver, heart, spleen, gall bladder and gonads) were examined for macroparasites following a standardised protocol. The taxonomic consistency of the identification was ensured thorough the entire study.

**Results:**

We discuss some problems in parasite identification, outline the composition of the parasite faunas in cod in the six North East Atlantic regions, provide novel data on parasite prevalence and abundance and a comparative assessment of the structure of the regional parasite faunas with respect to the higher-level taxonomic groupings, host specificity and zoogeographical distribution of the parasites. Altogether 57 different parasite forms were found including seven new host records (*Diclidophora merlangi*, *Rhipidocotyle *sp., *Fellodistomum *sp., *Steringotrema *sp., *Cucullanus *sp., *Spinitectus *sp., and *Chondracanthus ornatus*). The predominant groups of cod parasites were trematodes (19 species) and nematodes (13 species) including larval anisakids which comprised 58.2% of the total number of individuals.

**Conclusion:**

Our study reveals relatively rich regional parasite faunas in cod from the North East Atlantic which are dominated by generalist parasites with Arcto-Boreal distribution. Further, it provides more detailed data on the distribution in the North East Atlantic of the majority of cod parasites which may serve as baselines for future studies on the effect of climate change. Based on the faunal comparisons, predictions can be made in relation to the structure and diversity of the parasite communities in the North East Atlantic regions studied.

## Background

The Atlantic cod, *Gadus morhua *L. (Teleostei: Gadidae) is one of the most important commercial fish species along the eastern and western coasts of the North Atlantic and due to this our knowledge of its life history and ecology surpasses that of most other fish species (see [1] for a review). Cod acts as intermediate, paratenic or definitive host to a large number of parasite species (107 taxa, reviewed by Hemmingsen & MacKenzie [2]). The long list of cod parasites summarised by these authors illustrates the omnivorous nature of its diet, and its complex role within the food webs. Numerous studies on cod parasites and/or host-parasite interactions have been conducted on both sides of the North Atlantic [2]. A sizeable part of these studies focuses on various aspects of the population variability (geographical, seasonal, genetic) of anisakid nematodes which have drawn wide attention because cod hosts species of considerable economic and medical importance [3-18].

Other parasitic groups studied due to their pathological effects on cod include copepods [19,20], monogeneans [21,22], myxozoans [23] and protozoans [24]. Another group of studies is associated with taxonomy/faunistic aspects of some cod parasites: digeneans [25-33]; monogeneans [21,34]; and acanthocephalans [35-39]. A further aspect of studies on cod parasites that provides faunistic data is their use as biological markers of fish populations both in the North West Atlantic [8,40-47] and North East Atlantic regions [35,48-53].

Overall, the literature on cod parasites reveals both, differential study effort and an uneven geographical coverage. On the other hand, comparative analyses on local cod parasite faunas are virtually lacking, a series of studies by Hemmingsen and colleagues carried out off Northern Norway being a notable exception [11,54,55]. The present study was carried out within the framework of the multidisciplinary international project CODTRACE aimed at combining different traceability techniques to establish spawning and harvest location of *G. morhua*. It profited from the parasitological examination of large samples of cod collected during 2002–2003 from six regions of the North East Atlantic which yielded abundant baseline data on the distribution and abundance of macroparasites of this host in the study areas.

Here we outline the composition of the macroparasite faunas in cod in the six North East Atlantic regions studied which add to the most recent checklist of cod parasites of Hemmingsen & MacKenzie [2] especially with respect to parasite geographical distribution. We provide novel data on species prevalence and abundance and a comparative assessment of the structure of the regional parasite faunas with respect to the higher-level taxonomic groupings, host specificity and zoogeographical distribution of the parasites.

## Materials and Methods

### Cod samples

**Figure 1:**
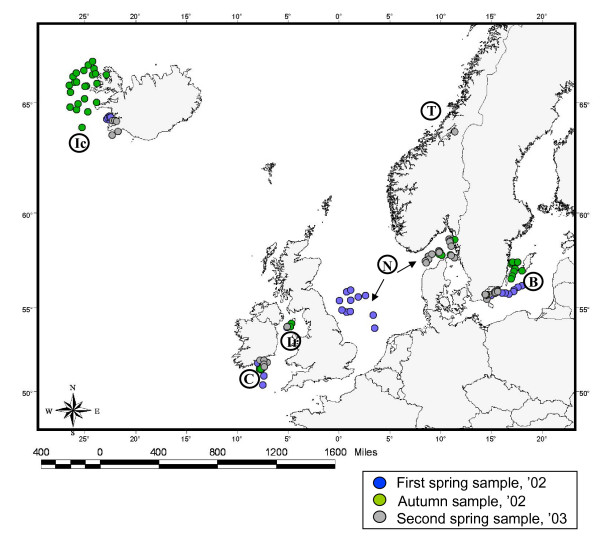
**Sampling locations in the regions of study**. *Abbreviations*: B, Baltic Sea; C, Celtic Sea; Ic, Icelandic waters; Ir, Irish Sea; N, North Sea; T, Trondheimsfjord.

The study is based on 826 fish sampled in six regions of the NE Atlantic [Baltic, Celtic, Irish and North seas, Icelandic waters and Trondheimsfjord (Norway)] in 2002 (spring and autumn) and 2003 (spring). Fish represent six different stocks, according to the demarcation in management units of the International Council of Exploration of the Sea [56] (Figure 1; Table 1).

**Table 1:** Sample sizes by region (areas as currently demarcated by ICES) and season

**Sampling area**	**ICES area**	**Spring ** **2002**	**Autumn ** **2002**	**Spring ** **2003**	**Total**
**Baltic Sea**	Division IIId Subdivisions 25 and 27	59^a^	61^b^	60^a^	180
**Celtic Sea**	Division VIIg	23	56	59	138
**Icelandic waters**	Division Va	45	62	58	165
**Irish Sea**	Division VIIa	60	52	24	136
**North Sea**	Divisions IVb and IIIa	27^c^	60^d^	60^d^	147
**Trondheimsfjord**	Division IVa/NCC^e^			60	60

**Total**		214	291	321	826

^a^Subdivision 25; ^b^Subdivision 27; ^c^Division IVb; ^d^Division IIIa; ^e^NCC: Norwegian Coastal Cod stock

Fish were processed at the point of capture on scientific research vessels during groundfish surveys. Each fish was measured [total length (TL) and standard length (SL)] and weighted. Fish ranged from 16.5 to 119.5 cm in length (SL, mean 50 ± 16.5 cm) and from 0.045 to 15.3 kg in weight (mean 2.0 ± 2.2 kg). Visible parasites from the external and internal body surfaces were removed and preserved in 70% ethanol and the entire viscera including the gills of each fish were removed and stored frozen (-20°C). These were shipped to the University of Valencia for parasitological examination.

### Parasite samples

The gills and internal organs (oesophagus, stomach, intestine, pyloric caeca, liver, heart, spleen, gall bladder and gonads) were examined separately. Muscles were not available for examination. After collection of all parasite specimens, all organs were pressed individually between glass plates and further screened for parasites. Examination was carried out with the aid of high magnification stereomicroscope. All parasites were collected and preserved in 70% ethanol. Representative sub-samples of 30 specimens (when parasite numbers very high) or all specimens (when few) were prepared for detailed morphological examination and identification in Canada balsam mounts whereas the remaining specimens were identified in wet mounts. Trematodes, monogeneans and cestodes were stained with iron acetocarmine, dehydrated through an alcohol series, cleared in dimethyl phthalate and examined/identified as permanent mounts in Canada balsam. Nematodes and acanthocephalans were examined as wet mounts in saline solution or temporary mounts in clearing liquids (lactic acid or glycerine).

As a result of the sample processing skin and eyes were not subjected to examination and therefore the metacercariae [*Cryptocotyle lingua *(Creplin, 1825) and *Diplostomum spathaceum *(Rudolphi, 1819)] and cutaneous monogeneans of the genus *Gyrodactylus *were not collected. Furthermore, gill monogeneans of the latter genus were not detected probably due to their degradation during freezing. The abundance of the copepod *Holobomolochus confusus *from the nasal cavity of cod, as well as of some other external parasites such as hirudineans, amphipods and isopods may appear underestimated since the samples were collected in the field by non-specialists. Despite these sampling problems, the vast material of cod analysed in the present study was collected using identical sampling procedure in all regions. Parasitological examination was carried out following a standardised protocol and the taxonomic consistency of the identification was ensured thorough the entire study.

## Results

### Comments on parasite identification and taxonomy

The 171,821 metazoan parasites collected were identified to the lowest taxonomic level possible (most often to species level). The identification of some of the species found in this study is still controversial. Thus, Brinkman [57] pointed out that *Progonus muelleri *(Levinsen, 1881) has been confused with *Derogenes varicus *(Müller, 1774), the differences being the presence of a cyclocoel and the thicker egg shell in *P. muelleri*. Consequently many studies could have underestimated the presence of the latter species in cod. The material reported here as *D. varicus *agrees well with the morphology and metrical data for this species provided by Bray [58]. However, although *D. varicus *was the only species found in our study, an overestimation of its presence as in other studies is possible, especially in the cases of high parasite abundance where identification was based on representative sub-samples.

Another case of difficult identification is that of the species of the genus *Stephanostomum *Looss, 1899. Of the three species listed in cod by Hemmingsen & MacKenzie [2] one, *Stephanostomum baccatum *(Nicoll, 1907), belongs to group 1 of Bray & Cribb [59] [*i.e*. vitellarium anterior extent (% of hindbody devoid of follicles) < 10%] whereas the two other species, *S. caducum *(Looss, 1901) and *S. pristis *(Deslongchamps, 1824) belong to group 2 (*i.e*. vitellarium anterior extent > 10%). In *S. pristis *from *G. morhua *in Danish waters Køie [60] found that, in hundreds of adult specimens, the number of circumoral spines ranged from 2 × 18 to 2 × 26, with a dominance of specimens with 2 × 23–25 whereas the 2 × 18 arrangement was rarely found. She concluded that *S. caducum *(described with 2 × 25 oral spines) was probably a synonym of *S. pristis *(originally described with 2 × 18 spines). However, Bartoli & Bray [61] redescribed *S. pristis *on Mediterranean material and detected no variation in circumoral spine number. They concluded that two species were mixed in [60]. Similarly, Karlsbakk [62] found no variation in circumoral spine number in his specimens from *Enchelyopus cimbrius *and *G. morhua *from off western Norway. This author pointed out that the specimens he considered to be *S. caducum *from *Trisopterus esmarkii *and *Gadiculus argenteus thori *also show minimal variation in spine number, with 2 × 24–25 circum-oral spines. Both *S. pristis *and *S. caducum *were present in the total collection of cod parasites in this study. However, circumoral spines are very fragile and can be lost in frozen material or during the handling process. It was, therefore, impossible to identify at species level the numerous specimens in all samples; these are henceforth referred to as *Stephanostomum *spp.

A third species-identification problem concerns the pseudophyllidean cestode *Parabothrium gadipollachii *(Rudolphi, 1810) which is difficult to differentiate from *Abothrium gadi *van Beneden, 1871 (but see [63]). Although reported, the former species is infrequent in cod and common in other gadoids. The only adult cestode recovered in the present study was identified as *A. gadi *on the basis of its agreement with the morphological data of Williams [63]. Moreover, cross-sections confirmed the main distinctive feature at the generic level of the present material, *i.e*., vitelline follicles continuous throughout the length of the proglottis and intermingled with testes in *A. gadi *(*vs *partly cortical and partly medullary vitelline follicles, not intermingled with testes in *P. gadipollachii*, see [64]).

Larval stages pose a number of obstacles to parasite identification mostly because of their simple morphology and the fact that many of the species discriminating features are not yet present at the early stages of parasite development. One of the new host records, *Rhipidocotyle *sp., was found at a metacercarial stage and identified to generic level only. Larval anisakids represent a more complex case with respect to identification due to the limitations in using morphological characters [65-67] and the overall complicated taxonomy due to the presence of sibling species. Thus, *Anisakis simplex *(Rudolphi, 1809) is a complex of three sibling species with fairly similar morphology [68]. *A. simplex *A for which the name *A. pegreffii *was proposed, is mainly Mediterranean, whereas *A. simplex sensu stricto *(*s.s*.) (formerly differentiated as *A. simplex *B) has a North Atlantic distribution and *A. simplex *C was recently reported from the Pacific by Mattiucci *et al*. [69] (see also [70,71] and references therein). Although it is possible that the third-stage larvae (L3) of *Anisakis *collected in the present study belong to *A. simplex *(*s. s*.), (see [71] for a detailed review on hosts and distribution) a more conservative identification was adopted, designating the specimens to *A. simplex sensu lato *(*s. l*.).

*Pseudoterranova decipiens *(Krabbe, 1878), another anisakid species, also represents a species complex. Electrophoretic analyses of gene enzyme systems identified three sibling species: *P. decipiens *A, in grey seals (*Halichoerus grypus*) in the northeast Atlantic; *P. decipiens *B, in harbour seals (*Phoca vitulina*); and *P. decipiens *C, in bearded seals (*Erignathus barbatus*) [72,73]. The taxonomy of these species has recently been revised by Mattiucci & Nascetti [71] so they should be referred to as *P. krabbei *Paggi et al., 1878, *P. decipiens *(*s. s*.) and *P. bulbosa *Kobb, 1888, respectively. A further two taxa were also later included in the *P. decipiens *species complex: *Pseudoterranova azarasi *(Yamaguti & Arima, 1942) and *P. cattani *George-Nascimento & Urrutia, 2000 [71]. Among the sibling species of the *P. decipiens *(*s.l*.) complex, present in the North East Atlantic waters, only two, *P. decipiens *(*s. s*.) and *P. krabbei *have been reported as parasites of *G. morhua *from the North East Atlantic (see [71] for a detailed host-parasite list).

Similarly, Nascetti *et al*. [74] in a multilocus electrophoretic study, recognised three species within *Contracaecum osculatum *(Rudolphi, 1802) species complex: *C. osculatum *A, occurring mainly in bearded seals in the eastern and western North Atlantic; *C. osculatum *B, found mainly in phocid seals in the eastern and western North Atlantic; and *C. osculatum *C, found in the grey seal *Halichoerus grypus *from off Iceland and Baltic Sea [71]. Orecchia *et al*. [75] added two other species, named *C. osculatum *D and *C. osculatum *E, found as adults in Weddell seals (*Leptonychotes weddellii*) in the Antarctic. Unfortunately, the above distinctions cannot be applied to the abundant larval collection in the present study due to the lack of reliable morphological features to discriminate the sibling species within *P. decipiens *and *C. osculatum*. Therefore, these forms were designated as *P. decipiens sensu lato *(*s. l*.) and *C. osculatum sensu lato *(*s. l*.).

The larval specimens *Hysterothylacium *(Ward & Magath, 1917) in the present collection were assigned to two species. Most third-stage (L3), and all fourth-stage larvae (L4) and adults, were identified as *H. aduncum *(Rudolphi, 1802) after Berland [76] and Anderson [70], whereas the L3 forms found spirally encysted in the submucosa of the digestive tract were identified as *H. rigidum *(Rudolphi, 1809) [77]. Morphological observations in the course of this study lead to the suggestion that the larval nematodes reported as *Spiruroideorum *"larvae" of Janiszewska [78] by Palm *et al*. [79] actually represent L3 larva of *H. rigidum*.

All cuculanid nematodes were identified as *Cucullanus cirratus *Müller, 1777 except for one individual found in the stomach. The specimen was somewhat damaged which precluded its identification to the species level. However, its small oral capsule indicated that it represents a form different from both *C. cirratus *and *Cucullanus heterochrous *Rudolphi, 1802. *Cucullanus *sp. was, therefore, considered a new host record. The three additional new host records, namely *Spinitectus *sp., *Fellodistomum *sp. and *Steringotrema *sp. were only identified to the generic level due to the scarcity of the material and the problematic taxonomy of the respective groups. However, our study represents the first record of representatives of the above genera in cod [2].

The most commonly reported adult acanthocephalan in cod, *Echinorhynchus gadi *Zoega in Müller, 1776, which has also been reported in a large number of other teleost species [80], was found to actually represent a species complex comprising three distinct, partially sympatric species in the NE Atlantic, all occurring in cod [39,81]. Since the distinction of these species was inferred from molecular evidence, we were not able to discriminate *E. gadi *which is further referred to as *E. gadi sensu lato *(*s. l*.).

### General description of the parasite fauna of cod

All fish were infected, except for three fish from the Baltic Sea. Species composition, prevalence and mean abundance of each parasite species in each region are summarized in Additional file 1 which also includes data on parasite specificity and distribution. The classification of the species with respect to the latter characteristics are based on the divisions and data of Hemmingsen & MacKenzie [2] to which the data compiled from the literature and the Host-Parasite Database at the Natural History Museum, London [80] were added. Specificity and distribution categories were only assigned to parasites identified to species level.

Altogether 57 different parasite forms were found. Of these, 41 were identified to species level (including two species which were not considered true parasites of cod, see below), 12 were identified to generic level whereas five larval forms (one digenean, four cestodes and the copepod larvae which certainly represented juvenile stages of the copepod species recovered) were identified to family/order (Additional file 1). Nine species were found for the first time in cod in the present study: the monogenean *Diclidophora merlangi *(Kuhn, in Nordmann, 1832) (described in [34]); the trematodes *Rhipidocotyle *sp., *Fellodistomum *sp. and *Steringotrema *sp.; the larval cestode *Schistocephalus gasterostei *Fabricius, 1780; the adult nematodes *Cucullanus *sp. and *Spinitectus *sp. and the copepods *Acanthochondria soleae *(Krøyer, 1838) and *Chondracanthus ornatus *Scott, 1900. Two of these cannot be considered true parasites of cod but rather part of the fish food content, namely the copepod *A. soleae *which was recovered from the stomach of one fish in a rather decomposed state and *S. gasterostei *which was found in the body cavity of a three-spined stickleback *Gasterosteus aculeatus *from the stomach content of one cod. The latter two species (labelled 'D' in Additional file 1) were excluded from all comparisons. *G. morhua *is therefore a new host record for seven species recovered in the present study.

The predominant groups of cod parasites were trematodes (19 species) and nematodes (13 species). The other higher taxonomic groups had low representation: 8 cestodes, 7 copepods, 3 acanthocephalans, 2 hirudineans, 1 monogenean, 1 isopod and 1 amphipod. Overall, 60% of the species were represented by adult parasites. However, more than half of the parasite individuals (58.8%) were larval forms. The majority of the larvae were anisakid nematodes which comprised 58.2% of the total number of individuals. Data in Additional file 1 reveal an overall high variation in the prevalence and abundance of the 55 parasite forms between the six study regions. Eleven species were present in all regions [the trematode *Lepidapedon elongatum *(Lebour, 1908); the nematodes *A. simplex *(*s. l*.), *C. osculatum *(*s. l*.), *H. aduncum*, *H. rigidum*, *P. decipiens *(*s. l*.), *Ascarophis crassicollis *Dollfus & Campana-Rouget, 1956 and *Capillaria gracilis *(Bellingham, 1840); and the acanthocephalans *Corynosoma semerme *(Forssell, 1904), *C. strumosum *(Rudolphi, 1802) and *E. gadi *(*s. l*.)]. Three species [*H. aduncum*, *Derogenes varicus *(Müller, 1784) and *A. simplex *(*s. l*.)] showed high prevalence and abundance in the overall collection (prevalence 83.9%, 65.6% and 53.4%, respectively; mean abundance 31.6, 21.8 and 85.3, respectively). In contrast, ten species infected only one fish each.

### Structure of the regional parasite faunas in cod: Higher-level taxa

**Figure 2:**
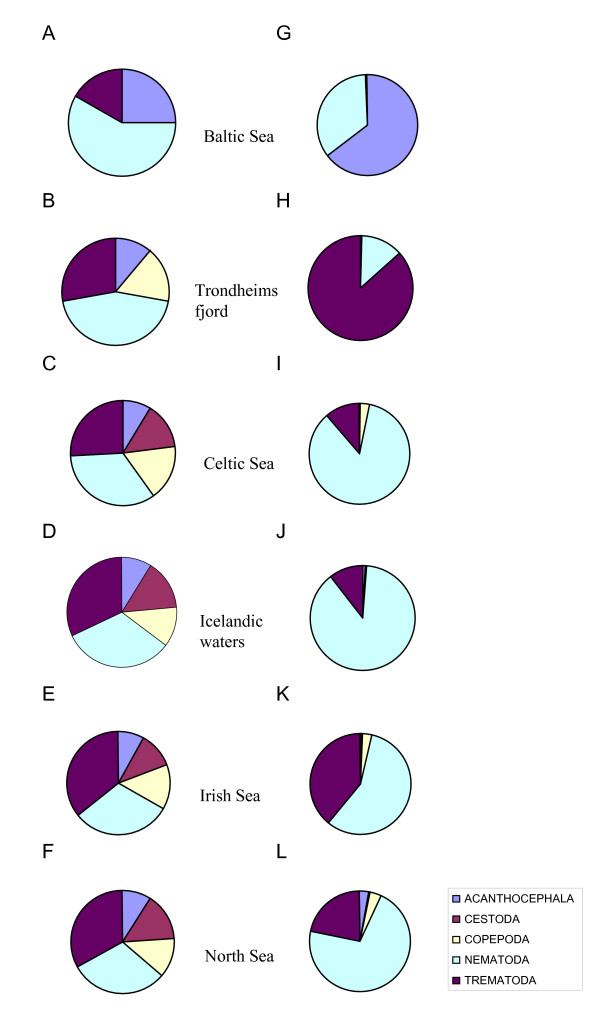
Taxonomic structure of the parasite faunas of *G. morhua *in the six North East Atlantic regions with respect to relative species richness (A-F) and relative abundance (G-L) of the higher parasite taxa.

The maximum number of parasite species (37) was found in the collection from the Celtic Sea, followed by those from Icelandic waters and the Irish and North seas (36 species each). Species richness of the parasite faunas of cod was substantially lower in Trondheimsfjord and the Baltic Sea (18 and 12 species, respectively). The higher-level taxonomic structure of the parasite faunas in cod from the 6 regions is graphically represented in Figure 2. The figure shows the relative representation in terms of both number of species and individuals of the major parasite taxonomic groupings in cod, namely, Trematoda, Cestoda, Nematoda, Acanthocephala and Copepoda. The four remaining higher-level taxonomic groups, *i.e*. Monogenea, Hirudinea, Amphipoda and Isopoda, were poorly represented, both in terms of species and individuals, and were omitted for clarity.

The species representation was very similar in all regions except for the Baltic Sea and Trondheimsfjord where nematodes represented a distinctly higher proportion of all species and cestodes were absent (Figure 2A,B). The four other regional faunas (*i.e*. in cod from Celtic, Irish and North seas and Icelandic waters) exhibited similar richness of the higher taxa groupings (Figure 2C–F). The above two distinct faunas also showed marked differences with respect to the relative abundance of the higher taxonomic groups. Although nematodes were the richest taxon in the Baltic Sea collection, the high numerical dominance of acanthocephalans was the most distinctive trait of the cod parasite fauna in this region. Three species [*E. gadi *(*s. l*.), *C. semerme *and *C. strumosum*] represented nearly 64% of all parasite individuals in the Baltic Sea collection (Figure 2G). On the other hand, Trondheimsfjord fauna was distinct in the exceptionally high relative abundance of trematodes (86.4%, see Figure 2H) which was mainly due to two species with similar high prevalence and abundance, *L. elongatum *and *Lepidapedon rachion *(Cobbold, 1858), which accounted for 67% and 19% of all individuals, respectively. Another characteristic feature of the Trondheimsfjord fauna was the low representation of larval nematodes and the numerical dominance of adult nematodes. Thus, two species, *C. gracilis *and *C. cirratus *(accounting for 7% and 5%, of the total parasite number, respectively) represented 53.2% and 41.8%, of all nematodes in this collection, respectively.

Overall, the taxonomic structure of the regional parasite faunas based on the relative abundance of the higher taxa (Figure 2G–L) differed substantially from that based on species richness (Figure 2A–F). A much higher representation of nematodes was revealed in the four regions which exhibited similarity with respect to the species richness structure of the faunas (*i.e*. Celtic, Irish and North seas and Icelandic waters). These could be grouped in pairs with respect to the relative abundance of the numerically dominant taxa: (i) the faunas of Celtic Sea and Icelandic waters which exhibited substantially elevated numbers of nematode individuals (Figure 2I,J); and (ii) the faunas of Irish and North seas which had higher proportions of trematode individuals (Figure 2K,L).

Nematodes represented over 85% of individual parasites in the Celtic Sea collection. The most representative species were *A. simplex *(*s. l*.), *H. aduncum *and *C. osculatum *(*s. l*.) which comprised 42%, 24% and 5%, respectively, of the total nematode abundance. Trematodes were less abundant when compared to the faunas of the second group (*i.e*. Irish and North seas, 11% of all parasites). *D. varicus, H. communis *Odhner, 1905 and *Stephanostomum *spp. accounted for 8%, 2% and 1% of all parasite individuals, respectively and represented 74.7%, 15.9% and 7% of all trematodes. The parasite fauna of cod in Icelandic waters was very similar to that of the Celtic Sea in terms of proportions of nematodes and trematodes (88 and 11% of all individuals, respectively). The most abundant nematodes were (as in the Celtic Sea collection) *A. simplex *(*s. l*.), *H. aduncum *and *C. osculatum *(*s. l*.) which comprised 61%, 12% and 10%, respectively, of all individuals. The most abundant trematode species in the Icelandic waters fauna was *D. varicus *which represened 10% of all individuals.

The cod parasite fauna of the Irish Sea was characterised by the higher numerical representation of trematodes (39%) and a comparatively low relative abundance of nematodes which comprised 57% of all parasites (Figure 2K). The trematodes *D. varicus*, *H. communis *and *Stephanostomum *spp. comprised 29%, 7% and 2% of all parasites, respectively. Although the most widespread and abundant nematode species were roughly the same as in the Celtic and Icelandic faunas, their proportions of the total abundance differed. *H. aduncum*,*C. osculatum *(*s. l*.), *Ascarophis morrhuae *Van Beneden, 1871 and *A. simplex *(*s. l*.) accounted for 31%, 9%, 7% and 4% of all parasites, respectively.

The parasite fauna of the North Sea cod could be considered intermediate between the Irish Sea and the other two (*i.e*. Celtic Sea and Icelandic regional faunas) with respect to the relative trematode abundance which accounted for nearly 22% of parasites. *D. varicus*, *H. communis *and *Stephanostomum *spp. accounted for 13%, 4% and 4%, respectively, of all forms in the North Sea collection whereas nematodes comprised 70% of all parasite forms. Again, the most common and abundant species were almost the same as in the Irish Sea fauna, but their relative proportions differed. The nematodes *A. simplex *(*s. l*.), *H. aduncum*, *A. crassicollis *and *C. osculatum *(*s. l*.) represented 29%, 24%, 9% and 1% of all parasites, respectively.

**Figure 3:**
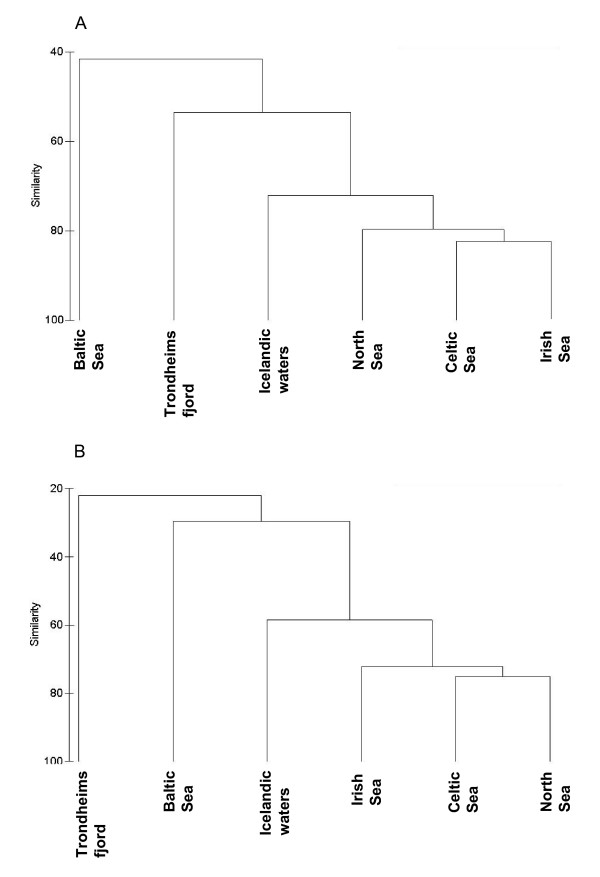
Cluster analysis dendrogram (group-average linkage) of the parasite faunas of *G. morhua *in the six North East Atlantic regions, using Bray-Curtis similarity matrices based on species prevalence (A) and mean abundance (B).

The above distinctions of the regional parasite faunas with respect to the higher taxonomic level structure translated into a similar but more refined picture at the species level, as revealed by a cluster analysis using the similarity matrix (Bray-Curtis similarity) based on species prevalence (Figure 3A) and abundance (Figure 3B). The major differences between regions observed at the higher taxonomic level appeared valid in the species similarity analysis in that the Baltic Sea and Trondheimsfjord faunas were most dissimilar whereas the four other regions formed a cluster at high similarity levels (72.2% and 58.5% for matrices based on prevalence and mean abundance, respectively). However, the grouping within the latter differed from that described above on the basis of the higher-level parasite representation. The faunas of North, Celtic and Irish seas formed a group at high similarity levels (79.7% and 72.2% for matrices based on prevalence and mean abundance, respectively) whereas the parasite fauna of cod in Icelandic waters was somewhat distinct and occupied an intermediate position between this group and the Trondheimsfjord/Baltic Sea faunas. The latter two joined at low similarity levels (prevalence data 53.6% and 41.6%; abundance data 29.6% and 21.9%, respectively).

### Structure of the regional parasite faunas in cod: Host specificity of parasites

The classification of Hemmingsen & MacKenzie [2] was generally followed with respect to host specificity of cod parasites. No host specific parasites were found in this study. In fact, very few specific parasites are recognized in this host species [2]. Therefore two main groups are recognised here: (i) parasite species reported from cod and other gadoid fish species (gadoid specialists, labelled 'GS' in Additional file 1); and (ii) parasite species reported from a wider range of host species (generalists, labelled 'G' in Additional file 1). Hemmingsen & MacKenzie [2] considered an additional 'accidental species' category (labelled 'A' in Additional file 1) but stressed that this is an arbitrary decision and that 'accidental species' can also be placed in the 'generalist species' category due to their low specificity behaviour. This suggestion was followed in the present study for the species not listed by the latter authors.

**Figure 4:**
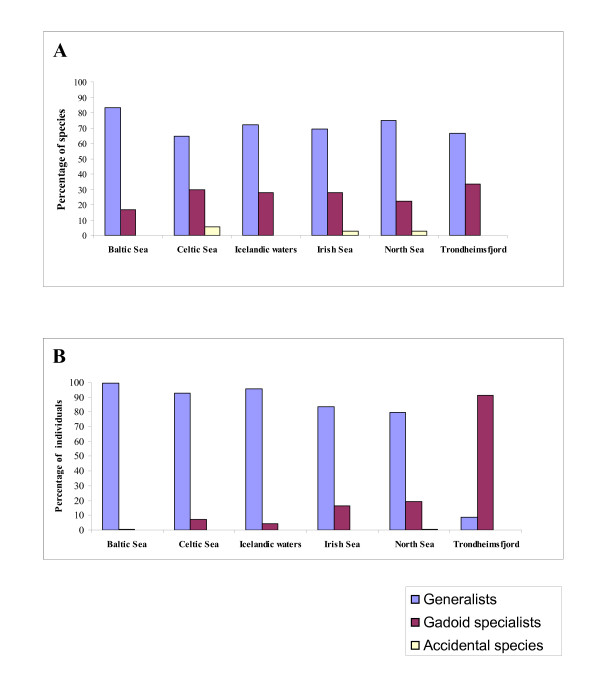
Relative richness (A) and abundance (B) structure of the parasite faunas of *G. morhua *in the six North East Atlantic regions with respect to host specificity of the parasites.

Generalist parasites comprised the majority of the cod parasite fauna (40 species which accounted for 85% of all individuals) whereas gadoid specialist were poorly represented (12 species, 15% of all parasite individuals). The three species considered as accidental made up a minute proportion of the species and individuals. The structure of the six regional faunas in terms of parasite specificity illustrated in Figure 4 revealed that generalist species dominate over gadoid specialists with respect to both relative richness and abundance. This dominance was most expressed in the fauna of the Baltic Sea where only two gadoid specialist species (16.7% of the species) were present (the trematode *L. elongatum *and the nematode *A. crassicollis*. These also had very low abundance (0.4% of all individuals). The parasite faunas of the other low salinity region, Trondheimsfjord, exhibited an opposite pattern, *i.e*. the highest, especially with respect to abundance, representation of the gadoid specialists' category (Figure 4). Six species of this category, the trematodes *L. elongatum *and *L. rachion*, the nematodes *A. morrhuae *and *C. cirratus*, and the copepods *Clavella adunca *(Strøm, 1762) and *Holobomolochus confusus *(Stock, 1953), represented 33.3% of the species and 91.3% of the individuals found in this collection.

The other four regions (Celtic, Irish and North seas and Icelandic waters) showed similar proportions and shared the same gadoid specialist species. Seven gadoid specialists were common for the four regions: *L. elongatum*, *Stephanostomum *spp., *A. gadi*, *A. morrhuae*, *A. crassicollis*, *C. cirratus *and *C. adunca*. Three species, *Prosorynchoides gracilescens *(Rudolphi, 1819), *L. rachion *and *Ascarophis filiformis *Polyansky, 1952 were present in the parasite faunas of cod from Celtic and Irish seas and Icelandic waters but absent in the North Sea fauna. However, gadoid specialists were numerically better represented in the Irish and North Sea faunas as compared to the Celtic Sea and Icelandic waters faunas (16.4 and 19.5% of individuals *vs *7.2 and 4.3%, respectively, see Figure 4B).

### Structure of the regional parasite faunas in cod: Zoogeographical distribution of parasites

Although the zoogeographical groupings of Hemmingsen & MacKenzie [2] are rather general and related to the geographical distribution of cod only, their classification was followed to ensure the consistency of the comparisons. Parasite species were therefore split into three categories with respect to their geographical distribution: (i) Arctic-Boreal species (labelled 'A-B' in Additional file 1) which 'infect cod in the northern part of its distribution but not in southern warmer waters'; (ii) Boreal species (labelled 'B' in Additional file 1) whose distributions 'overlap that of cod and extend beyond it to more temperate southern waters'; and (iii) species of worldwide distribution (labelled 'W' in Additional file 1) which have been reported from many different parts of the world. The category 'not applicable' (labelled 'NA' in Additional file 1) refers to taxa not identified to species level.

Due to its wide geographical extent our study added new data on the distribution of most species infecting cod in the North Eeast Atlantic (Additional file 1). Among these is the new host record, the monogenean *D. merlangi *found in the Celtic and North seas. This species was also recorded in the Arctic (samples from the Natural History Museum, London examined by Perdiguero-Alonso *et al*. [34]; see also [82]), which leads us to consider its distribution as Arctic-Boreal following the definitions of Hemmingsen & MacKenzie [2]. The hemiurid *Hemiurus luehei *Odhner, 1905 (not reported by Hemmingsen & MacKenzie [2]) actually belongs to the Boreal group (found in Irish and North seas in our study, see also [83]). Hemmingsen & MacKenzie [2] considered that *A. simplex *(*s. s*.) which has only been reported from cod [69,71], has a northern hemisphere distribution (within the worldwide category) in both the Atlantic and Pacific Oceans. However, recent data suggesting that of the three sibling species only *A. simplex *B has a North Atlantic distribution [68] and the present data (see Additional file 1) suggest that the anisakid larvae in cod in the North East Atlantic exhibit an Arctic-Boreal distribution.

The hirudinean *Johanssonia arctica *(Johansson, 1899) was considered a low Arctic species. Although we found this species in cod from Icelandic waters only, data by Appy & Dadswell [84] suggest that it has an Arctic-Boreal distribution. The copepod species recovered in cod for the first time in our study, *C. ornatus*, appears to have a worldwide distribution (see [85]) whereas the isopode *Gnathia elongata *(Krøyer, 1847) should be considered an Arctic-Boreal species (see also [86]). On the other hand, the present study confirmed the classification of six species as Boreal by Hemmingsen & MacKenzie [2]: *Prosorhynchoides gracilescens *(Rudolphi, 1819), *Lepidapedon rachion *(Cobbold, 1858), *Opechona bacillaris *(Molin, 1859), *A. gadi*, *H. rigidum *and *C. strumosum*; these were also found in Icelandic waters in the course of the study.

**Figure 5:**
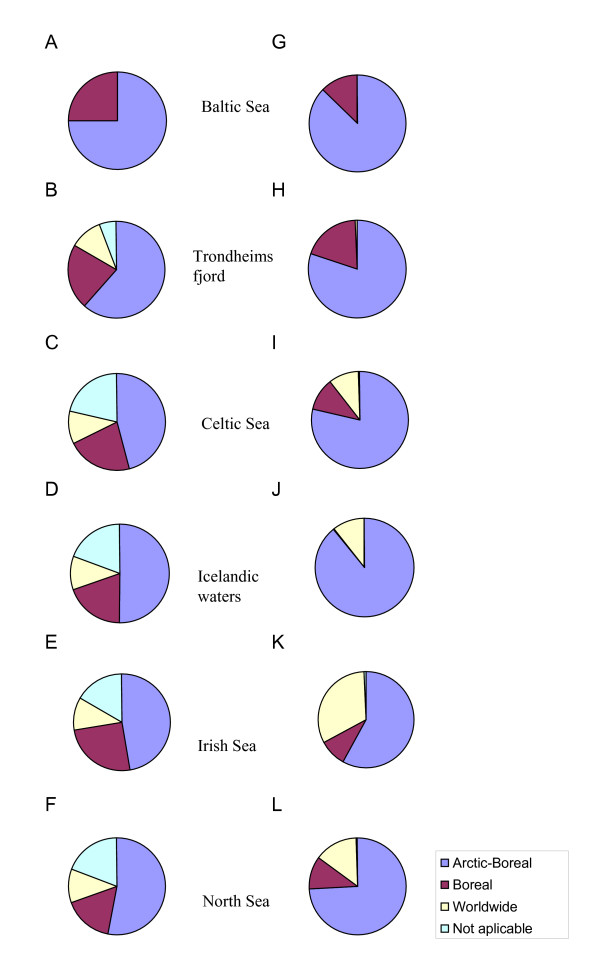
Relative richness (A-F) and abundance (G-L) structure of parasite faunas of *G. morhua *in the six North East Atlantic regions with respect to zoogeographical distribution of parasites.

In all regions, the best-represented group in terms of parasite species was the Arctic-Boreal (Additional file 1; Figure 5A–F). Roughly 20% of the species were Boreal, and the reminder had worldwide distribution except for the Baltic Sea fauna which lacked species of the latter category. The relative abundance of the species with Arctic-Boreal distribution was distinctly higher (Figure 5G–L). The latter group dominated in faunas of the Baltic Sea, Trondheimsfjord and Icelandic waters representing 87%, 80% and 89% of all individuals, respectively).

Of the 11 parasite species present in all regions studied, nine had an Arctic-Boreal distribution [*L. elongatum*,* A. simplex *(*s. l*.), *C. osculatum *(*s. l*.),*H. aduncum*, *P*. *decipiens *(*s. l*.), *C. gracilis*, *C. semerme *and *E. gadi *(*s. l*.)] and two species (*H. rigidum *and *C. strumosum*) had Boreal distributions. The relative abundance of the Boreal species was higher in the Trondheimsfjord fauna (19% of all individuals) than in the other regions (range 9–13% of all individuals) except for the parasite fauna of cod in Icelandic waters which showed a negligible proportion of this distribution category (0.1% of all individuals). The relative abundance of species with worldwide distribution was highest in the Irish Sea fauna (32% of all individuals). In contrast, Trondheimsfjord fauna had very few individuals with worldwide distribution (0.5% of all individuals) because of the presence of only two species of this group, *D. varicus *and *C. adunca*.

## Discussion

The list of 57 macroparasite taxa reported in the present study (Additional file 1) comprises nearly 56% of the parasites found in *G. morhua *throughout its distributional range (a total of 97 species, resulting from compilation of data gathered as early as 1932 from both North West and North Eeast Atlantic, see [2]). It indicates a high regional richness of the metazoan parasites of cod in the North East Atlantic. These results conform to the diverse and non-selective diet of cod, its wide depth distribution and migratory behaviour. It is also possible that increased sampling effort has contributed to the high diversity of the parasite list reported here and this is supported by the seven new host records. Of these, only the gadoid specialist *D. merlangi*, which mainly parasitises whiting (*Merlangius merlangus *(L.), see [82]) belongs to the Arctic-Boreal category. The newly recorded helminth species mainly belong to generalist genera with a wide geographical distribution whereas the copepod *Chondracanthus ornatus *is typical of calionomid perciforms [85]. The geographical distribution of the Callionymidae overlap with that of cod, thus its recovery indicates some interaction with calionomids.

However, the regional parasite faunas of cod exhibited a generally lower richness (63–65% of the total list) with a notable decrease in the Baltic Sea and Trondheimsfjord (21 and 32%, respectively). Parasites from all metazoan taxa were recorded in the present study, with eleven species present in all regions. The predominant higher taxa in the regional faunas in terms of number of species were trematodes and nematodes. However, the taxonomic structure of the faunas based on the relative abundance of the higher taxa revealed that nematodes (mostly anisakid larvae) represent the majority of all parasite individuals. This fact can be related to cod being a voracious predator, and with a long life-span, which facilitates larval accumulation).

The regional faunas exhibited differences with respect to both higher-level taxonomic structure and species-level comparisons. Generally, the fauna of the brackish-water regions [Baltic Sea (7–13.6‰) and Trondheimsfjord (10–33‰)] differed substantially from those in the high-salinity regions (Celtic, Irish and North seas and Icelandic waters, salinity range 34.2–35.4‰). Although variation in fish size between regions may have contributed to the observed variability, the much lower species richness recorded in the former two regions agrees with the lower salinity conditions that restrict the distribution and richness of the invertebrate fauna and consequently limiting the diversity of successful parasite life-cycles [87,88]. Remarkably, none of the species characteristic of low salinity and freshwater distributions reported previously in cod (*i.e. Podocotyle angulata *(Dujardin, 1845), *Raphidascaris acus *(Bloch, 1779), *Acanthocephalus lucii *(Müller, 1777), *Echinorhynchus salmonis *(Müller, 1784), *Neoechinorhynchus rutili *(Müller, 1780) and *Pomphorhynchus laevis *(Zoega in Müller, 1776) [2,87,89]) was recorded in our collections from the Baltic Sea and Trondheimsfjord.

On the other hand, although both regional faunas consisted of marine parasite species, their structure differed from that of the faunas from the open water regions in: (i) the poorer numerical representation of nematodes; (ii) the absence of cestodes; (iii) the absence or low abundance of species with worldwide distribution; and (iv) the composition with respect to host specificity categories [*i.e*. the strong numerical domination of generalists (Baltic Sea fauna) or gadoid specialist species (Trondheimsfjord fauna)]. These differences, therefore, indicate different transmission conditions in the two low-salinity regions. This suggestion is further reinforced by the notably different structure of the faunas in the latter regions characterised by the numerical dominance of generalist acanthocephalans [mostly *E. gadi *(*s. l*.), Baltic Sea] or gadoid specialist trematodes (*Lepidapedon *spp., Trondheimsfjord).

The overall prevalence of 88.3% of *E. gadi *(*s. l*.) observed in the present study agrees well with the high levels of infection in cod recorded in previous studies: 71.4% in the southern Baltic Sea [90] and 99.4% in the Bornholm Basin of Baltic Sea [37]. The mean intensities recorded here are similar (32.2 worms/host) to those in the latter study: 54.7 in smaller cod (21 to 30 cm body length) and 33.3 in larger cod (52 to 60 cm body length). Gammarid (*Gammarus oceanicus*) and caprellid (*Monoporeia femorata*) amphipods serve as intermediate hosts of *E. gadi *(*s. l*.) in the Baltic Sea [91]. Whereas the high infection levels in small cod may indicate that amphipods are an important component their diet, the heavy infection of large cod (> 61 cm; normally not feeding on amphipods) was explained by a transfer of parasites from prey fish to the large cod [37]. It is possible that both processes contribute to the infection of cod in the Baltic Sea collection since the size of the fish studied ranged from 31.4 to 89.6 cm (SL).

The dominance of trematodes in the Trondheimsfjord fauna reflects the highest infection levels of two *Lepidapedon *species (see comparative data in Additional file 1). Both species belong to the subfamily Lepocreadiinae of the Lepocreadiidae Odhner, 1905, which are found either in deep-sea fishes or in fishes from cold, shallow waters, most usually in gadiforms [30]. Whereas the present data on the overall prevalence of *L. elongatum *(60%) agree with previous observations in cod (up to 94.3% at various stations in Danish and adjacent waters [92]; up to 62% in juvenile (0+) cod [48,93-95]), *L. rachion *has so far been recovered at much lower prevalences in various locations in the North East Atlantic (range 3.3–20% *vs *45%, see [92]). Bray & Gibson [30] listed a wider range of final hosts (mostly gadoids) in the North East Atlantic for the latter species (*G. morhua, Melanogrammus aeglefinus, Merlangius merlangus, Pollachius pollachius, P. virens*, *Gymnacanthus tricuspis*, *Aspitrigla cuculus*). Trondheimsfjord is characterised by a rich fish fauna (16 gadiform species including 10 species of gadoids: *Gadiculus argenteus thori*, *G. morhua*, *M. aeglefinus*, *M. merlangus*, *Micromesistius poutassou*, *P. pollachius*, *P. virens*, *Trisopterus esmarki*, *T. minutus*, *Raniceps raninus*; the latter uncommon, J.A. Sneli pers. comm.) and this may explain the higher infection levels of *L. rachion *in this region. It is also possible that the dominance of the two *Lepidapedon *species in the parasite fauna in cod from Trondheimsfjord is related to the presence of conditions enhancing completion of their life-cycles. The life-cycle of *L. elongatum *was elucidated by Køie [25]. The rediae and cercariae develop in the gastropod *Onoba aculeus *and the metacercariae encyst in a variety of annelids; some may encyst in molluscs and echinoderms, but infections in these hosts are rare and probably short-lived [25]. The first intermediate host of *L. rachion *is believed to be *Nassarius reticulatus *and the metacercariae are said to occur in planktonic cnidarians, ctenophores, chaetognaths and polychaetes [26]. Sneli & Gulliksen [96] reported both intermediate hosts, *O. aculeus *and *N. reticulatus*, in Trondheimsfjord. However, the life-cycle of *L. rachion *has not apparently been completed experimentally. Bray & Gibson [30] considered the data on the second intermediate host puzzling, since the main final host of *L. rachion*, the haddock, *Melanogrammus aeglefinus *(L.), feeds as an adult almost entirely on benthic organisms. Nevertheless, cod studied at Trondheimsfjord were generally small-sized (SL range 16.5–48.0 cm) and it is possible that the proportion of small invertebrates in the diet of fish has contributed to the high representation of *Lepidapedon *spp.

Higher gadoid richness may also be associated with higher transmission rates which resulted in the dominance in the Trondheimsfjord fauna of the adult stages of two gadoid specialist nematodes, *C. cirratus *and *C. gracilis*. Final hosts of *C. cirratus *are Gadidae and Merluccidae, occasionally salmon, *Salmo salar*, see [97]). Although Anderson [70] suggests a direct infection of final host (by ingestion of free-living second-stage larvae, L2), calanoid (*Acartia *sp., *Centropages *sp., *Temora *sp.) and cyclopoid (*Oitona similis*) copepods and sand gobies, *Pomatoschistus minutus*, were found to serve as experimental intermediate hosts of *C. cirratus *[98]. Third-stage (L3) larva of *C. gracilis *hatch from the egg in the intestinal tract of either the intermediate fish host (sand goby, *P. minutus*; experimental data) or an invertebrate transport (paratenic) host [99]. Køie's [98] data, based on examination of 350 naturally infected cod (8–78 cm long), support this suggestion. She found that group 1 and older cod contained L3-stage larvae, intermediate stages and adult worms of *C. cirratus*, indicating that they could become infected throughout the year; however the pattern of infection suggested that cod over 20 cm long became infected mainly in summer by eating infected fish (including smaller cod). It is possible that the high infection levels with *C. cirratus *and *C. gracilis *in cod from Trondheimsfjord originate from ingestion of sand gobies which are common in the region.

One of the main results of the present study was the overall higher structural similarity of the parasite faunas in cod from Celtic, Irish and North seas and Icelandic waters, perhaps due to the similar oceanographic characteristics of these four regions. The domination of the generalist Arctic-Boreal anisakid nematodes [*A. simplex *(*s. l*.), *C. osculatum *(*s. l*.) and *H. aduncum*] represented a characteristic feature of the four regional faunas. *A. simplex *(*s. l*.) and *C. osculatum *(*s. l*.)] utilise marine mammal predators of cod [2,71] as final hosts and follow a similar life history pattern.

Adult *A. simplex *(*s.l*.) have been reported in a large number of cetaceans and pinnipeds [71,100]. Eggs passed by marine mammals embryonate to the L2-stage larvae in sea water. When ingested by marine crustaceans (e.g. euphasiids, copepods) they develop to the L3 stage. Teleosts become infected by ingesting the first intermediate hosts see [70] and references therein. Klimpel *et al*. [101] studied the life-cycle of *A. simplex *in the northern North Sea and found that one copepod and four euphasiid species served as obligatory intermediate hosts. These authors revealed an obligatory second intermediate host, *Maurolicus muelleri *(Sternoptychidae), and stated that piscivorous (*Pollacius virens, Melanogrammus aeglefinus, Etmopterus spinax*) and planktivorous and juvenile fishes (*Clupea harengus, Trisopterus esmarki, Melanogrammus aeglefinus*) serve as paratenic hosts of *A. simplex*. Although the data on the life-cycle of *C. osculatum *species complex are somewhat wanting [70] copepods may appear important as intermediate hosts [70,102]).

Klimpel *et al*. [101] and Klöser *et al*. [103] suggested that *A. simplex *and *C. osculatum*, respectively, are able to utilise fish host species that are available in a given locality. This versatile behaviour coupled with the vagility of the final hosts, may explain the wide distribution and abundance of these species. *H. aduncum *possesses an even more resourceful life-cycle. Final hosts of this species are numerous predaceous teleosteans (clupeids, gadids, salmonids and others, see [97]). Third stage larvae develop in *Acartia tonsa *and harpacticoid copepods, various amphipods, isopods and mysids [104]. The latter can also serve as second intermediate hosts [105]. Furthermore, ctenophores, chaetognaths, polychaetes and ophiuroids which become infected by ingesting infected crustaceans, may act as obligatory intermediate hosts or paratenic (transport) hosts [104,106].

Despite their overall structural similarity, the four faunas could be grouped in two pairs, those from Celtic Sea and Icelandic waters *vs *those from the Irish and North seas. It appears that the grouping with respect to the higher trematode representation in cod parasite faunas in Irish and North seas (*vs *Celtic Sea and Icelandic fauna) is related to the sampling locations. Thus, the fauna from deeper and ocean influenced locations in the Celtic Sea and Icelandic waters were dominated by nematodes whereas the more coastal and shallower locations (in the Irish and North seas) exhibited higher proportions of trematode individuals.

Overall, generalist parasites with Arctic-Boreal or worldwide distribution comprised the best represented group of the cod parasite fauna with respect to both richness and numerical dominance (due to the presence of anisakid nematodes). This finding supports the conclusion of Hemmingsen & MacKenzie [2] that cod acts as a distribution agent of generalist parasites in the North Atlantic because of its omnivorous diet, migratory behaviour and the mixture of stocks.

## Conclusion

To summarise, our study reveals relatively rich and abundant regional macroparasite faunas in cod from the North East Atlantic which are generally dominated by generalist parasites with Arctic-Boreal distribution. Furthermore, it provides more detailed data on the distribution in the North East Atlantic of the majority of cod parasites which may serve as baselines for future studies on the effect of climate change on parasite distribution in abundance since the regions occupied by cod are expected to experience some of the largest anthropogenic climate changes in the world. The higher-level faunal comparisons suggest that differences may exist in the feeding behaviour between cod sampled in the six regions. On the other hand, the composition of the regional faunas may be determined largely by variations in the abundance of the intermediate hosts. These suggestions are supported by the high regional variation in the prevalence and abundance of the parasite species which translated into somewhat different clustering pattern based on similarity at the species level. Finally, based on the above comparisons, the following predictions can be made in relation to the structure and diversity of the parasite communities:

(i) Parasite communities in cod from the Baltic Sea and Trondheimsfjord would show much lower richness, abundance, diversity and would exhibit higher variation in composition and structure.

(ii) Parasite communities in cod from the other four regions (Irish, Celtic and North seas and Icelandic waters) would have the highest richness, abundance, diversity and similarity and would be dominated by larval nematodes.

(iii) With 11 species (nearly a fifth of the total number) shared between the six regions there would be a substantial homogenisation in the composition of both the component and infracommunities. *A. simplex *(*s. l*.), *H. aduncum *and *D. varicus *would contribute substantially to the structural homogeneity between communities.

## Competing interests

The authors declare that they have no competing interests.

## Authors' contributions

DPA and FEM carried out the parasitological examination of fish and the identification of the parasites. DPA performed the comparisons and drafted the manuscript. JAR and AK contributed to the design of the study, assisted in data interpretation and helped to draft the manuscript. All authors read and approved the final manuscript.

## Supplementary Material

Additional file 1 Host specificity, distribution, prevalence (P), mean abundance (MA ± SD), median abundance (M, shown if >0 only) of parasites in *G. morhua* from the Baltic, Celtic, Irish and North seas, Icelandic waters and Trondheimsfjord (Norway). New host records are marked with an asterisk. *Abbreviations for host specificity categories*: D: food content; G, generalist; GS, gadoid specialist.*Abbreviations for distribution categories*: A-B, Arctic-Boreal; B, Boreal; W, worldwide; NA, Not applicable. Click here for file
