# Supplementary material for: Composition and structure of the parasite faunas of cod, Gadus morhua L. (Teleostei: Gadidae), in the North East Atlantic
Source: Parasit Vectors. 2008 Jul 18;1:23. doi: 10.1186/1756-3305-1-23 (PMC2503959; doi:10.1186/1756-3305-1-23)
Supplement: Additional file 1 — Host specificity, distribution, prevalence (P), mean abundance (MA ± SD), median abundance (M, shown if >0 only) of parasites in G. morhua from the Baltic, Celtic, Irish and North seas, Icelandic waters and Trondheimsfjord (Norway). New host records are marked with an asterisk. Abbreviations for host specificity categories: D: food content; G, generalist; GS, gadoid specialist.Abbreviations for distribution categories: A-B, Arctic-Boreal; B, Boreal; W, worldwide; NA, Not applicable. [file 1756-3305-1-23-S1.doc]

**Additional file 1.** Host specificity, distribution, prevalence (P), mean abundance (MA) ± SD and median abundance (M, shown if >0 only) of parasites in *Gadus morhua* from the Baltic, Celtic, Irish and North seas, Icelandic waters and Trondheimsfjord (Norway). New host records are marked with an asterisk. *Abbreviations for host specificity categories*: D: food content; G, generalist; GS, gadoid specialist. *Abbreviations for distribution categories*: A-B, Arctic-Boreal; B, Boreal; W, worldwide; NA, Not applicable.

| **Region**  **(sample size)** |  |  | **Baltic Sea**  **(n = 180)** | **Celtic Sea**  **(n = 138)** | **Icelandic waters**  **(n = 165)** | **Irish Sea**  **(n = 136)** | **North Sea**  **(n = 147)** | **Trondheimsfjord**  **(n = 60)** |
| --- | --- | --- | --- | --- | --- | --- | --- | --- |
| **Fish standard length (range in cm)** | |  | **31.4-89.6** | **25-95.5** | **30.5-119.5** | **27.3-75** | **22.3-109.5** | **16.5-48** |
| **Parasite species** | **Host specificity** | **Distribution** | **P (%)**  **MA ± SD**  **(M)** | **P (%)**  **MA ± SD**  **(M)** | **P (%)**  **MA ± SD**  **(M)** | **P (%)**  **MA ± SD**  **(M)** | **P (%)**  **MA ± SD**  **(M)** | **P (%)**  **MA ± SD**  **(M)** |
| **MONOGENEA** |  |  |  |  |  |  |  |  |
| *Diclidophora merlangi* | GS | A-B |  | 0.7  0.01 ± 0.09 |  |  | 0.7  0.01 ± 0.08 |  |
| **TREMATODA (metacercariae)** | |  |  |  |  |  |  |  |
| Bucephalinae gen. sp. | G | NA |  | 8.7  0.12 ± 0.52 | 1.2  0.01 ± 0.11 | 12.5  0.93 ± 5.53 | 2.0  0.08 ± 0.70 |  |
| *Cryptocotyle lingua* | G | A-B |  |  |  |  | 1.4  0.01 ± 0.12 |  |
| *Otodistomum* sp. | G | NA |  |  | 1.8  0.02 ± 0.13 |  | 2.7  0.34 ± 2.46 |  |
| *Prosorynchoides gracilescens* | GS | B |  | 2.9  0.09 ± 0.71 | 0.6  0.01 ± 0.16 | 0.7  0.01 ± 0.17 |  |  |
| *Prosorhynchus crucibulum* | G | NA |  | 5.8  0.09 ± 0.39 |  | 2.9  0.03 ± 0.17 |  | 1.7  0.02 ± 0.13 |
| * *Rhipidocotyle* sp. | G | NA |  |  |  | 0.7  0.01 ± 0.09 |  |  |
| **TREMATODA (adult forms)** | |  |  |  |  |  |  |  |
| *Derogenes varicus* | G | W |  | 85.5  15.05 ± 23.34  (6) | 86.1  54.85 ± 155.91  (10) | 100  34.92 ± 43.29  (20.5) | 87.1  14.83± 38.23  (4) | 30.0  0.48 ± 0.93 |
| * *Fellodistomum* sp. | G | NA |  |  | 0.6  0.01 ± 0.08 |  |  |  |
| *Gonocerca phycidis* | G | W |  | 10.1  0.14 ± 0.44 |  | 23.5  1.07 ± 4.08 | 0.7  0.01 ± 0.08 |  |
| *Hemiurus communis* | G | B | 0.6  0.01 ± 0.07 | 49.3  3.20 ± 10.29 |  | 75.0  8.40 ± 26.00  (2) | 48.3  4.73 ± 20.29 | 50.0  1.90 ± 3.12  (0.5) |
| *Hemiurus levinseni* | G | A-B |  |  | 7.9  0.34 ± 1.67 |  |  |  |
| *Hemiurus luehei* | A | B |  |  |  | 5.2  0.10 ± 0.44 | 10.9  0.78 ± 4.04 |  |
| *Lecithaster* sp. ?*gibbosus* | G | A-B |  |  |  | 0.7  0.03 ± 0.34 | 0.7  0.02 ± 0.25 |  |
| *Lepidapedon elongatum* | GS | A-B | 2.2  0.18 ± 1.7 | 0.7  0.01 ± 0.09 | 9.7  1.72 ± 11.21 | 5.9  0.17 ± 0.84 | 2.0  0.21 ± 1.88 | 60.0  177.47 ± 585.76  (2) |
| *Lepidapedon rachion* | GS | B |  | 2.2  0.06 ± 0.43 | 1.8  0.05 ± 0.42 | 2.2  0.05 ± 0.39 |  | 45.0  49.42 ± 191.24 |
| *Opechona bacillaris* | G | B |  |  | 0.6  0.01 ± 0.08 | 0.7  0.01 ± 0.09 |  |  |
| *Podocotyle reflexa* | G | A-B |  |  | 6.1  0.08 ± 0.38 |  |  |  |
| *Stephanostomum* spp. | GS | B |  | 21.0  1.41 ± 5.53 | 3.6  0.07 ± 0.40 | 29.4  1.85 ± 7.39 | 40.1  4.69 ± 14.68 |  |
| * *Steringotrema* sp. | G | NA |  |  |  |  | 0.7  0.01 ± 0.08 |  |
| **CESTODA (larval forms)** |  |  |  |  |  |  |  |  |
| *Grillotia* sp. | G | NA |  |  |  |  | 0.7  0.03 ± 0.33 |  |
| *Hepatoxylon* sp. | G | NA |  | 1.5  0.03 ± 0.27 |  |  |  |  |
| *Lacistorhynchus* sp. | G | NA |  | 2.9  0.11 ± 0.79 |  | 5.2  0.06 ± 0.27 | 2.0  0.02 ± 0.14 |  |
| *Scolex pleuronectis* | G | W |  |  | 0.6  0.01 ± 0.08 | 2.2  0.02 ± 0.15 |  |  |
| Pseudophyllidea fam. gen. sp. | G | NA |  | 2.2  0.04 ± 0.25 | 0.6  0.01 ± 0.08 |  |  |  |
| * *Schistocephalus gasterostei* | D |  | 0.6  0.02 ± 0.30 |  |  |  |  |  |
| Trypanorhyncha fam. gen. sp. | G | NA |  | 4.4  0.06 ± 0.31 | 1.2  0.01 ± 0.11 | 5.2  0.07 ± 0.30 | 3.4  0.14 ± 1.06 |  |
| Unidentified plerocercoids | G | NA |  |  | 0.6  0.01 ± 0.08 |  | 1.4  0.02 ± 0.18 |  |
|  |  |  |  |  |  |  |  |  |
| **CESTODA (adult forms)** |  |  |  |  |  |  |  |  |
| *Abothrium gadi* | GS | B |  | 16.7  0.21 ± 0.50 | 3.6  0.04 ± 0.19 | 22.1  0.31 ± 0.66 | 5.4  0.05 ± 0.23 |  |
| **NEMATODA (larval forms)** |  |  |  |  |  |  |  |  |
| *Anisakis simplex* (*s.l.*) (L3) | G | A-B | 15  2.37 ± 13.25 | 92.0  74.89 ± 134.59  (19.5) | 99.4  327.29 ± 433.06  (121) | 36.0  4.65 ± 42.41 | 50.3  34.33 ± 127.02  (1) | 5.0  0.05 ± 0.22 |
| *Contracaecum osculatum* (*s.l.*) (L3) | G | A-B | 53.9  6.84 ± 16.07  (1) | 72.5  8.19 ± 15.50  (2) | 94.6  56.62 ± 62.85  (34) | 62.5  10.94 ± 23.40  (2) | 15.7  1.29 ± 5.73 | 8.3  0.23 ± 0.96 |
| *Hysterothylacium aduncum* (L3) | G | A-B | 41.7  1.83 ± 5.49 | 68.8  11.28 ± 20.80  (3.5) | 97.6  37.9 ± 35.71  (28) | 72.8  11.85 ± 20.67  (3.5) | 78.2  11.95 ± 39.37  (4) | 65.0  1.20 ± 1.47  (1) |
| *Hysterothylacium rigidum* (L3) | G | B | 40.6  6.34 ± 51.17 | 52.2  14.57 ± 36.15  (1) | 6.7  0.37 ± 2.47 | 8.1  0.20 ± 0.95 | 23.1  1.33 ± 7.19 | 8.3  0.12 ± 0.42 |
| *Pseudoterranova decipiens* (*s.l.*) (L3) | G | A-B | 3.9  0.07 ± 0.39  (7) | 51.5  2.23 ± 4.22  (1) | 52.7  3.51 ± 12.47  (1) | 22.1  0.86 ± 3.00 | 12.9  1.38 ± 9.77 | 1.7  0.02 ± 0.13 |
| *Rhapidascaris* sp. (L3) | G | NA |  |  |  |  | 0.7  0.01±0.08 |  |
| **NEMATODA (adult forms)** |  |  |  |  |  |  |  |  |
| *Ascarophis morrhuae* | GS | A-B |  | 20.3  1.11 ± 4.83 | 52.1  11.96 ± 32.49  (1) | 66.9  7.96 ± 20.64  (2) | 20.4  1.01 ± 3.78 | 1.7  0.02 ± 0.13 |
| *Ascarophis crassicollis* | GS | A-B | 1.1  0.01 ± 0.11 | 26.8  2.20 ± 11.49 | 1.8  0.03 ± 0.23 | 45.6  3.38 ± 8.08 | 31.3  11.39 ± 37.92 |  |
| *Ascarophis filiformis* | GS | A-B |  | 0.7  0.01 ± 0.09 | 7.9  0.18 ± 1.01 | 2.9  0.03 ± 0.17 |  |  |
| *Capillaria gracilis* | G | A-B | 3.9  0.13 ± 0.76 | 7.3  0.10 ± 0.41 | 32.7  1.14 ± 4.15 | 1.5  0.01 ± 0.12 | 4.8  0.09 ± 0.44 | 88.3  18.25 ± 38.34  (8) |
| *Cucullanus cirratus* | GS | A-B |  | 71.0  4.80 ± 9.29  (2) | 79.4  6.74 ± 9.21  (3) | 53.7  3.38 ± 10.29  (1) | 55.1  3.24 ± 7.21  (1) | 93.3  14.32 ± 19.43  (8) |
| * *Cucullanus* sp. | G | NA |  | 0.7  0.01 ± 0.09 |  |  |  |  |
| *Hysterothylacium aduncum* | G | A-B | 13.9  0.36 ± 1.28 | 94.2  31.67 ± 40.02  (19) | 87.3  25.78 ± 33.40  (14) | 79.4  25.69 ± 38.41  (13.5) | 85.7  16.33 ± 35.28  (6) | 5.0  0.12 ± 0.58 |
| * *Spinitectus* sp. | G | NA |  | 5.1  0.13 ± 0.90 | 1.2  0.01 ± 0.11 | 1.5  0.02 ± 0.19 |  |  |
| **ACANTHOCEPHALA (post-cystacanths)** | |  |  |  |  |  |  |  |
| *Corynosoma semerme* | G | A-B | 10.6  0.36 ± 1.28 | 3.6  0.04 ± 0.24 | 0.6  0.01 ± 0.16 | 0.7  0.01 ± 0.09 | 0.7  0.01 ± 0.08 | 1.7  0.02 ± 0.13 |
| *Corynosoma strumosum* | G | B | 6.1  0.14 ± 0.73 | 18.8  0.36 ± 1.02 | 7.3  0.09 ± 0.35 | 14.0  0.26 ± 1.26 | 14.3  1.24 ± 6.33 |  |
| **ACANTHOCEPHALA (adult forms)** | |  |  |  |  |  |  |  |
| *Echinorhynchus* *gadi* (*s.l.*) | G | A-B | 88.3  32.24 ± 44.79  (16) | 5.1  0.16 ± 1.30 | 53.9  4.55 ± 9.87  (1) | 12.5  0.29 ± 1.42 | 28.6  2.35 ± 9.02 | 30.0  0.48 ± 1.02 |
| **HIRUDINEA (adult forms)** |  |  |  |  |  |  |  |  |
| *Calliobdella nodulifera* | G | A-B |  |  |  | 0.7  0.01 ± 0.09 | 2.0  0.02 ± 0.14 |  |
| *Johanssonia arctica* | G | A-B |  |  | 0.6  0.01 ± 0.08 |  |  |  |
| **COPEPODA (larval forms)** |  |  |  |  |  |  |  |  |
| *Caligus* sp. copepodite | G | NA |  |  |  | 0.7  0.01 ± 0.09 |  |  |
| Copepoda fam. gen. sp. copepodite | G | NA |  | 0.7  0.01 ± 0.09 |  |  |  |  |
| **COPEPODA (adult forms)** |  |  |  |  |  |  |  |  |
| * *Acanthochondria soleae* | D |  |  |  |  | 0.7  0.01 ± 0.09 |  |  |
| *Caligus curtus* | G | A-B |  | 1.5  0.01 ± 0.12 | 3.6  0.06 ± 0.38 | 16.2  0.65 ± 2.97 | 9.5  0.27 ± 1.56 |  |
| *Caligus diaphanus* | A | B |  | 0.7  0.01 ± 0.09 |  |  |  |  |
| *Caligus elongatus* | G | A-B |  | 24.6  2.04 ± 5.35 | 13.3  0.60 ± 3.84 | 16.9  0.43 ± 1.69 | 20.4  1.68 ± 6.44 |  |
| * *Chondracanthus ornatus* | A | W |  | 0.7  0.01 ± 0.09 |  |  |  |  |
| *Clavella adunca* | GS | W |  | 72.5  2.96 ± 3.88  (2) | 56.4  2.06 ± 3.45  (1) | 67.7  2.74 ± 5.22  (2) | 71.4  2.36 ± 3.95  (1) | 43.3  0.92 ± 1.41 |
| *Holobomolochus confusus* | GS | B |  |  |  |  |  | 1.7  0.02 0.13 |
| *Lernaeocera branchialis* | G | A-B |  | 6.5  0.09 ± 0.37 | 19.4  0.30 ± 0.68 | 4.4  0.04 ± 0.21 | 17.0  0.30 ± 0.96 | 21.7  0.33 ± 0.73 |
| **AMPHIPODA** |  |  |  |  |  |  |  |  |
| *Lafystius sturionis* | G | A-B |  |  |  |  | 0.7  1.27 ± 15.42 |  |
| **ISOPODA** |  |  |  |  |  |  |  |  |
| *Gnathia elongata*  (praniza larva) | G | A-B |  | 0.7  0.01 ± 0.09 | 1.8  0.02 ± 0.19 |  |  |  |
